# Openness influences emotion recognition in middle-aged and older adults through hippocampal volume and fluid intelligence: a serial mediation model

**DOI:** 10.3389/fpubh.2026.1847936

**Published:** 2026-08-13

**Authors:** Yan Wang, Libin Song, Pang Jin, Bo Zhou

**Affiliations:** 1 Department of Clinical Psychology, The Third Hospital of Quzhou, Quzhou, China; 2 Department of Science and Education, The Third Hospital of Quzhou, Quzhou, China; 3 Department of Psychiatric Rehabilitation, The Third Hospital of Quzhou, Quzhou, China; 4 Department of Psychiatry, The Third Hospital of Quzhou, Quzhou, China

**Keywords:** cognitive aging, emotion recognition, fluid intelligence, hippocampal volume, openness, serial mediation

## Abstract

**Background:**

Openness to experience is positively associated with emotion recognition ability in middle-aged and older adults, yet the underlying neurocognitive mechanisms remain unclear.

**Methods:**

Using a serial mediation model, the present study examined the neurocognitive pathways through which openness to experience influences emotion recognition accuracy and processing speed via hippocampal volume and fluid intelligence in 912 middle-aged and older adults aged 36–100 years, with age, sex, intracranial volume, and acquisition site included as covariates.

**Results:**

Openness had a significant direct positive effect on emotion recognition accuracy. The indirect effect of openness through fluid intelligence was significant and accounted for 85.4% of the total indirect effect. The serial indirect pathway through hippocampal volume followed by fluid intelligence was also significant. The same serial pathway was replicated for processing speed. Notably, hippocampal volume additionally showed a significant direct path to processing speed, a finding absent in the accuracy model.

**Conclusion:**

These findings reveal a serial neurocognitive pathway from personality trait to brain structure, through domain-general cognitive resources, and ultimately to social cognitive performance. Fluid intelligence appears to be the primary neurocognitive mechanism linking openness-related hippocampal preservation to emotion recognition performance, and the significant serial pathway further suggests that hippocampal volume may not directly affect emotion recognition, but rather exerts its effect indirectly through fluid intelligence. The results suggest that individuals with higher openness showed larger hippocampal volume, which in turn was associated with better fluid intelligence and greater emotion recognition accuracy. The replication of this pathway for processing speed, together with a novel direct hippocampal contribution to response speed, provides converging support for the robustness of the proposed neurocognitive mechanism across complementary performance indicators.

## 1 Introduction

The global population age structure continues to shift toward older age groups. Life expectancy increased from 49.0 years in 1950 to 71.7 years in 2021, and the aging ratio rose continuously between 2000 and 2021 in 188 of 204 countries and territories (1). Population aging is becoming increasingly prevalent globally, and one of its core features is a decline in social cognition (2). Facial emotion recognition, a core subdimension of social cognition (3), likewise shows marked decline in middle and later adulthood (4). Declining emotion recognition may impair individuals’ accurate interpretation of others’ emotional experiences and, in turn, affect real-world social adaptation (5). Identifying the factors associated with individual differences in emotion recognition among middle-aged and older adults and clarifying their underlying mechanisms are therefore important for understanding social cognitive aging. Emotion recognition is a central component of nonverbal communication, because affective information is often conveyed through facial expression, eye contact, body posture, and movement. Difficulties in recognizing others’ emotional expressions may therefore impair everyday communication and have been linked to interpersonal problems, reduced social competence, poorer interpersonal functioning, and lower quality of life (6–9). Given that social engagement and social relationships have important consequences for health and well-being in later life (10, 11), understanding factors associated with preserved emotion recognition is relevant not only to cognitive aging research but also to healthy aging and later-life social functioning.

Among the many factors that influence emotion recognition, personality traits have attracted attention because of their stability across time. In the Big Five model, openness reflects an individual’s receptiveness to new experiences, new ideas, and aesthetic experiences (12). Research indicates a stable positive association between openness and emotion recognition ability. Terracciano et al. (13) found that individuals higher in openness performed better on a facial emotion recognition task. Moreover, a cross-sectional study spanning ages 18 to 84 years found that openness made an independent contribution to emotion recognition, and this contribution remained robust after controlling for age, indicating that the effect of openness on emotion recognition is not merely a by-product of age-related decline but an age-independent personality effect (14). Another study using a dynamic multimodal emotion recognition task showed that emotion recognition ability was significantly positively associated with openness and was also related to empathy and emotion understanding, suggesting that the effect of openness on emotion recognition may be embedded in a broader network of socioemotional abilities (15). These findings suggest that openness may be an important predictor of individual differences in emotion recognition among middle-aged and older adults. However, the mechanisms through which openness influences emotion recognition remain to be clarified. Given that openness is also closely related to brain structural integrity (16) and multiple cognitive functions including fluid intelligence and executive function (17, 18), its influence may involve a multilevel transmission process from neural structure to cognitive processing.

Hippocampal volume is a potential neural structural basis linking openness and emotion recognition. Giannakopoulos et al. (19) conducted a 54-month longitudinal study in 65 older adults that included structural brain MRI, amyloid PET, and APOE genotyping, and found that individuals with higher openness scores showed significantly less left hippocampal atrophy during follow-up, suggesting that openness may serve as a protective factor for hippocampal integrity in aging. In addition, the prospective autopsy study by Terracciano et al. (20) suggested a possible association between personality traits and Alzheimer’s disease-related neuropathological burden. According to Braak staging theory (21), the spread of tau pathology from the entorhinal cortex to the hippocampal region is a critical step in neurodegeneration, suggesting that personality-related modulation of neuropathological processes may indirectly influence hippocampal preservation. Cognitive reserve theory provides further explanation for this protective effect by proposing that an active and socially integrated lifestyle helps strengthen the brain’s resistance to degenerative change (22), a proposition also supported by epidemiological evidence (23). At the same time, individuals higher in openness tend to maintain richer activity engagement and greater activity diversity (24), suggesting that openness may indirectly protect hippocampal volume by enhancing cognitive reserve. On the other hand, the hippocampus is one of the regions showing the most pronounced atrophy in middle and later adulthood (25). As a core structure in the medial temporal emotion-processing network, it is closely structurally and functionally connected with social brain regions such as the amygdala and fusiform gyrus. Using structural MRI, Szymkowicz et al. (26) found that larger hippocampal volume was significantly associated with faster correct identification of facial emotions in older adults, indicating that preservation of hippocampal volume may help maintain facial emotion recognition performance in aging. Openness may therefore indirectly support the maintenance of emotion recognition by protecting hippocampal volume.

Fluid intelligence represents a potential cognitive-processing basis linking openness and emotion recognition. On the one hand, multiple longitudinal studies indicate a protective effect of openness on cognitive function. The Openness-Fluid-Crystallized-Intelligence model proposed by Ziegler et al. (17) theoretically argues that individuals higher in openness are more likely to actively seek cognitively stimulating environments, and that sustained environmental enrichment slows the age-related decline in fluid intelligence. Multiple longitudinal studies provide empirical support for this view. Using the Aberdeen Birth Cohort, Hogan et al. (18) found that higher openness directly predicted maintenance of fluid reasoning and also indirectly supported maintenance of crystallized intelligence by promoting activity engagement. Jackson et al. (27) further demonstrated in an older sample that activity diversity is an important mediator of the association between openness and cognitive ability. On the other hand, emotion recognition is not a purely affective process; rather, it is a perceptual-cognitive integration task with substantial cognitive resource demands, involving visual encoding, pattern matching, and rapid categorical decision-making, all of which overlap with core processes of fluid intelligence. The meta-analysis by Schlegel et al. (28), based on 133 independent samples and 471 effect sizes, confirmed a robust positive association between emotion recognition ability and general intelligence, and this association was observed across fluid, crystallized, and spatial abilities. Large-sample factor-analytic work on the Penn CNB indicated that the social cognition factor to which emotion recognition belongs is moderately to strongly correlated with the executive and complex cognition factor that includes nonverbal reasoning, indicating that emotion recognition and fluid intelligence belong to different cognitive domains but share substantial cognitive-processing foundations (3). Fluid intelligence and working memory have also been shown to independently predict emotion recognition performance, and the age effect on emotion recognition remains significant after controlling for general cognition, suggesting that one part of age-related decline in emotion recognition accompanies decline in fluid intelligence, whereas another part reflects independent deterioration of the social cognitive system itself (29). Openness may therefore indirectly support emotion recognition by maintaining fluid intelligence, although emotion recognition likely also has other upstream determinants.

The close association between hippocampal volume and fluid intelligence provides a structural bridge integrating the two pathways above. Positive associations between hippocampal volume and fluid intelligence have been well documented in aging research, and this association appears age-specific. Reuben et al. (30) found that the positive correlation between hippocampal volume and fluid intelligence existed only in older adults, not in younger adults, suggesting that hippocampal atrophy becomes a key neural structural bottleneck constraining fluid intelligence in later life. O’Shea et al. (31) further found in a community sample of older adults that smaller hippocampal volume was significantly associated with lower NIH Toolbox Fluid Cognition Composite scores across multiple subdomains, including episodic memory, working memory, processing speed, and executive function. These findings theoretically connect the protective effect of openness on the hippocampus with the supportive effect of fluid intelligence on emotion recognition into a potential serial pathway. However, existing studies have examined these links separately. Although the associations between openness and hippocampal volume, openness and fluid intelligence, and fluid intelligence and emotion recognition have each received empirical support, no study has simultaneously integrated personality trait, brain structure, general cognition, and social cognition within a single model. Moreover, it remains untested whether hippocampal volume affects emotion recognition directly or only indirectly through fluid intelligence. Finally, the cognitive protective effect of openness has previously been demonstrated mainly in fluid intelligence and memory; whether it can extend to emotion recognition, a social cognitive indicator, through identifiable neurocognitive pathways remains to be tested.

**Figure 1:**
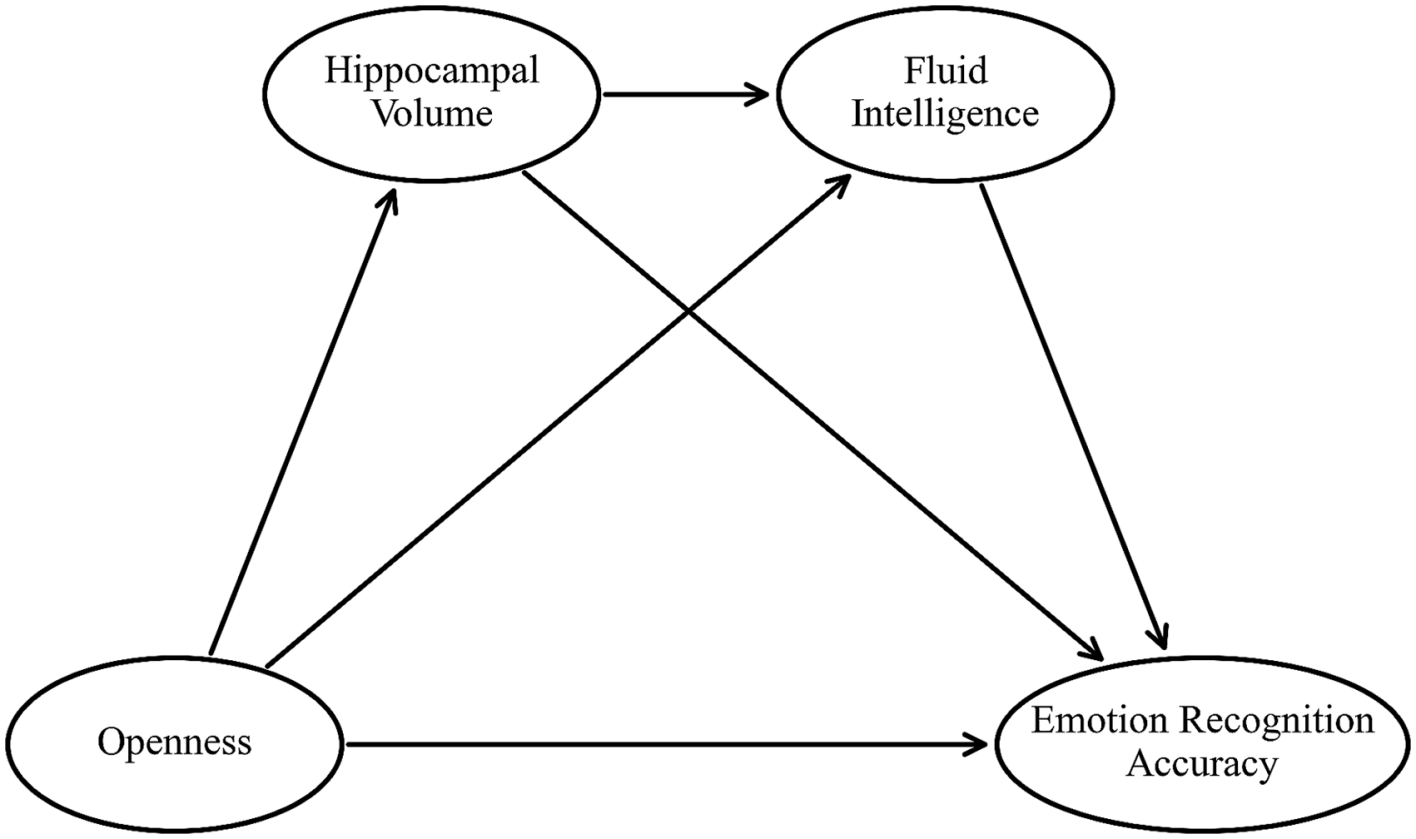
Theoretical serial mediation model. The diagram depicts the proposed pathways through which openness to experience influences emotion recognition accuracy in middle-aged and older adults via hippocampal volume and fluid intelligence. Age, sex, intracranial volume, and acquisition site are controlled as covariates in all structural equations but are not shown.

In summary, openness as a personality trait may influence emotion recognition in middle-aged and older adults through a serial pathway involving preservation of hippocampal volume and maintenance of fluid intelligence. Using large-sample cross-sectional HCP-Aging data, the present study tested the following hypotheses with a serial mediation model (32). Hypothesis 1: openness significantly and positively predicts the number of correct emotion-recognition responses and processing speed. Hypothesis 2: hippocampal volume mediates the association between openness and emotion recognition, such that openness indirectly influences emotion recognition by protecting hippocampal volume. Hypothesis 3: fluid intelligence mediates the association between openness and emotion recognition, such that openness indirectly influences emotion recognition by maintaining fluid intelligence. Hypothesis 4: hippocampal volume and fluid intelligence serially mediate the association between openness and emotion recognition, such that openness indirectly influences emotion recognition sequentially through hippocampal volume and fluid intelligence. The theoretical model of the present study is shown in Figure 1.

## 2 Methods

### 2.1 Participants

**Figure 2:**
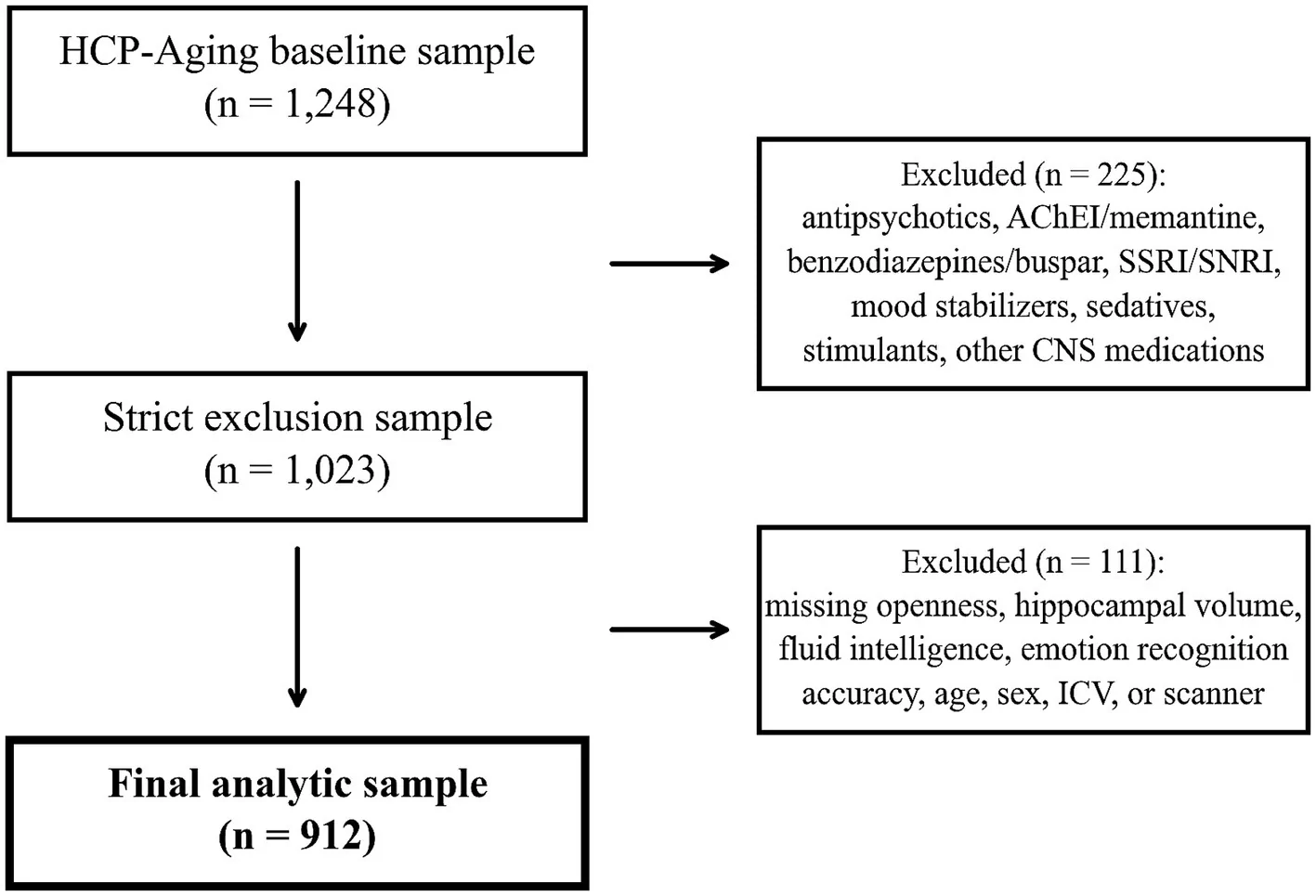
Participant inclusion and exclusion flowchart.

This study used the Human Connectome Project-Aging (HCP-A) dataset. Participants were recruited across four sites in the United States as part of the HCP-A study [Bookheimer et al. (33)]. This study was approved by the research ethics board of each participating institution and was conducted in accordance with the Declaration of Helsinki. All participants provided written informed consent. The present study is a secondary analysis of de-identified, openly released data and did not require additional ethics approval. The initial baseline sample included 1,248 participants. To reduce the potential confounding effects of psychotropic medications on brain structure and cognitive function, the present study applied strict exclusion criteria. Participants were excluded if they were currently taking any of eight classes of CNS-active medications, namely antipsychotics, acetylcholinesterase inhibitors/memantine, benzodiazepines/buspar, SSRI/SNRI, mood stabilizers, sedatives, stimulants, and other CNS medications, or if information on use of these medications was missing. After this screening step, 1,023 participants remained. On this basis, participants with missing data on any of the following variables were further excluded: openness, hippocampal volume, fluid intelligence, number of correct emotion-recognition responses, and the covariates age, sex, intracranial volume, and acquisition site. The final analytic sample consisted of 912 participants. The sample was racially and ethnically diverse: 69.7% White, 15.6% Black or African American, 7.5% Asian, 4.4% multiracial, and 2.5% other or unknown; 12.9% identified as Hispanic or Latino. In terms of educational attainment, participants had completed a mean of 17.41 years of education (SD = 2.26, range = 7–21). The sample selection flowchart is shown in Figure 2.

### 2.2 Measures and variables

#### 2.2.1 NEO five-factor inventory

The NEO Five-Factor Inventory (NEO-FFI) is a standardized self-report scale developed on the basis of Big Five personality theory (12). It includes five dimensions: neuroticism, extraversion, openness, agreeableness, and conscientiousness. The inventory contains 60 items, with 12 items per dimension, and uses a 5-point Likert scale. Sample items from the openness subscale include “I have a lot of intellectual curiosity,” “I have a vivid imagination,” and “I believe in the importance of art.” The scale has a mature five-factor measurement framework and has been widely used in personality assessment. In the present study, the openness score was used as the independent variable, reflecting an individual’s receptiveness to new experiences, new ideas, and aesthetic experiences. Higher scores indicate higher levels of openness.

#### 2.2.2 Structural MRI and hippocampal volume

Structural imaging data in HCP-A were acquired at four sites: the University of California, Los Angeles; the University of Minnesota; Massachusetts General Hospital; and Washington University in St. Louis. All sites used uniformly calibrated Siemens Prisma 3 T MRI scanners with 32-channel head coils to acquire T1-weighted images at 0.8 mm isotropic resolution (33). Imaging data were processed using the HCP Minimal Preprocessing Pipelines (34), which integrate the automated subcortical segmentation module in FreeSurfer 6.0 (35) for automated segmentation and volumetric measurement of whole-brain gray matter structures (36). Although all sites used the same scanner model and a unified acquisition protocol, subtle site-related systematic effects may still arise because of local calibration and physical environmental differences. Therefore, acquisition site was included in the analytic models as a covariate. Left and right hippocampal volumes were extracted from the FreeSurfer aseg output, measured in mm3, and their bilateral mean was used as the hippocampal volume index.

#### 2.2.3 NIH toolbox cognition battery

The NIH Toolbox Cognition Battery is a standardized cognitive assessment instrument developed with support from the U. S. National Institutes of Health and is intended to provide a unified measurement platform for cross-study comparisons of cognitive function (37). The battery includes two composites: fluid cognition and crystallized cognition. The Fluid Cognition Composite Score integrates performance on five subtests: the Flanker Inhibitory Control and Attention Test, the Dimensional Change Card Sort Test, the Picture Sequence Memory Test, the List Sorting Working Memory Test, and the Pattern Comparison Processing Speed Test, collectively reflecting performance in executive function, working memory, processing speed, and episodic memory (38). The fluid cognition composite used in the present study is a standardized score referenced to the age-35 group, calculated as the mean of standardized subtest scores. Higher values indicate better fluid cognitive performance. Because the present sample consisted of middle-aged and older adults and fluid cognition declines with age, the mean value of this index in the current sample was negative, reflecting cognitive performance relative to a young-adult reference group.

#### 2.2.4 Penn computerized neurocognitive battery

The Penn Computerized Neurocognitive Battery (Penn CNB) is a neurobehavioral assessment tool developed in conjunction with functional neuroimaging research and covers four cognitive domains, namely executive control, episodic memory, complex cognition, and social cognition (39). HCP-A used the Penn CNB to supplement social cognitive assessment not covered by the NIH Toolbox. In the present study, the Penn Emotion Recognition Test (ER-40) was used to assess facial emotion recognition ability. This test uses standardized facial emotion stimuli and requires participants to judge the emotional category expressed by each face. The total number of correct responses served as the primary measure of emotion-recognition accuracy, with higher scores indicating better performance. Reaction time was also recorded. To create a processing speed index, reaction time was multiplied by −1 prior to standardization, such that higher scores reflect faster responses. Factor-analytic research on the Penn CNB suggests that ER-40 belongs to the social cognition factor (3).

#### 2.2.5 Covariates

To control for potential confounding factors, age, sex, intracranial volume (ICV), and acquisition site were included as covariates in all analytic models. Age is a primary factor influencing brain structure and cognitive function, and sex, intracranial volume, and acquisition site may also confound structural brain measures and behavioral performance. Because intracranial volume serves as a correction for head size, it should be controlled in analyses involving regional brain volume. Acquisition site was entered as a set of dummy variables to account for possible systematic measurement differences across sites. Age and intracranial volume were standardized before entering the models, and sex was dummy coded. Sensitivity analyses additionally controlling for race, ethnicity, and years of education yielded consistent results (see Supplementary Tables S3, S4).

### 2.3 Statistical analysis

First, descriptive statistics were calculated for the demographic characteristics and core variables in the final analytic sample, and Pearson correlation coefficients were computed among the core variables.

Next, the serial mediation effect was tested using Model 6 in the PROCESS macro proposed by Hayes (32). In this model, openness was specified as the independent variable, hippocampal volume as the first mediator, fluid intelligence as the second mediator, and the number of correct emotion-recognition responses as the dependent variable. Processing speed was additionally examined as an alternative outcome in a parallel sensitivity analysis using the same model structure. All four core variables were standardized before entering the model to facilitate direct comparison of effect sizes across paths. Age, sex, intracranial volume, and acquisition site were controlled simultaneously in all equations of the model.

The significance of indirect effects was tested using bias-corrected bootstrap resampling with 5,000 resamples. Indirect effect estimates, bootstrap standard errors, and 95% confidence intervals were reported. An indirect effect was considered statistically significant when the 95% bootstrap confidence interval did not include 0. All analyses were conducted in R using the processR package.

## 3 Results

### 3.1 Descriptive statistics and correlation analysis

The final analytic sample included 912 participants, with a mean age of 60.45 years. Four hundred two were male and 510 were female. Descriptive statistics for the core variables and Pearson correlations among them are presented in Table 1. Detailed distribution plots of key variables are provided in Supplementary Figure S4.

**Table 1:** Descriptive statistics and zero-order Pearson correlations for all core variables (*N* = 912).

Variable	*M* (SD)/*n* (%)	1	2	3	4	5	6	7	8
1. Age (years)	60.45 (15.89)	–							
2. Sex	402 (44.1%)	0.041	–						
3. Openness	29.85 (5.92)	−0.086**	0.037	–					
4. Hippocampal volume ×10^3^ mm^3^	3.84 (0.47)	−0.555***	0.280***	0.160***	–				
5. Fluid intelligence	−0.48 (1.08)	−0.552***	−0.022	0.233***	0.410***	–			
6. ER-40 accuracy	33.55 (3.47)	−0.307***	−0.040	0.173***	0.242***	0.362***	–		
7. ER-40 processing speed	2479.81 (837.95)	0.566***	0.005	−0.075*	−0.390***	−0.570***	−0.281***	–	
8. Intracranial volume (mm^3^)	1522.56 (197.35)	0.241***	0.589***	0.075*	0.270***	−0.080*	−0.015	0.115***	–

M, mean; SD, standard deviation. Fluid intelligence = NIH Toolbox Fluid Cognition Composite Score standardized to a 35-year-old reference. This younger reference group was used because the NIH Toolbox norming procedure anchors scores to a peak performance baseline, such that negative scores reflect age-related decline relative to young adult norms. The use of a younger reference group follows the standardization protocol recommended for HCP-A fluid cognition data (33, 36).

ER-40 accuracy = number of correct responses (range 0–40). ER-40 processing speed (reaction time multiplied by −1 and standardized; higher scores = faster responses). Hippocampal volume = bilateral FreeSurfer mean.

*p < 0.05, **p < 0.01, ***p < 0.001.

Pearson correlation analyses showed that all core variables were significantly positively correlated. Specifically, openness was positively correlated with hippocampal volume (*r* = 0.160, *p* < 0.001), fluid intelligence (*r* = 0.233, *p* < 0.001), and the number of correct emotion-recognition responses (*r* = 0.173, *p* < 0.001). Hippocampal volume was positively correlated with fluid intelligence (*r* = 0.410, *p* < 0.001) and the number of correct emotion-recognition responses (*r* = 0.242, *p* < 0.001). Fluid intelligence was also positively correlated with the number of correct emotion-recognition responses (*r* = 0.362, *p* < 0.001). Processing speed showed a similar pattern of associations, being positively correlated with openness (*r* = 0.075, *p* = 0.024), hippocampal volume (*r* = 0.390, *p* < 0.001), fluid intelligence (*r* = 0.570, *p* < 0.001), and accuracy (*r* = 0.281, *p* < 0.001), indicating that faster responses were associated with higher scores on all core variables. This correlation pattern provides preliminary support for the theoretically proposed mediation relationships and lays the foundation for the subsequent serial mediation analysis.

### 3.2 Sex differences

Independent-samples t tests were conducted for each core variable to examine sex differences; the results are shown in Table 2. Hippocampal volume showed a significant sex difference, with males having significantly larger hippocampal volume than females [*t*(910) = 8.782, *p* < 0.001]. In contrast, openness [*t*(910) = 1.103, *p* = 0.270], fluid intelligence [*t*(910) = −0.662, *p* = 0.508], the number of correct emotion-recognition responses [*t*(910) = −1.206, *p* = 0.228], and processing speed [*t*(910) = 0.15, *p* = 0.881] did not differ significantly between males and females. Given the sex difference in hippocampal volume, sex was included as a covariate in the subsequent serial mediation models.

**Table 2:** Sex differences in core variables.

Variable	Male *M* (SD)	Female *M* (SD)	*t*	df	*p*
Openness	30.09 (5.90)	29.66 (5.94)	1.10	910	0.270
Hippocampal volume (mm^3^)	3.99 (0.48)	3.73 (0.43)	8.78	910	< 0.001
Fluid intelligence	−0.51 (1.04)	−0.46 (1.11)	−0.66	910	0.508
ER-40 accuracy	33.40 (3.39)	33.67 (3.53)	−1.21	910	0.228
ER-40 processing speed	2484.48 (714.53)	2476.13 (924.37)	0.15	910	0.881

Cohen’s *d* computed using pooled SD; positive values indicate Male > Female.

### 3.3 Serial mediation analysis

**Figure 3:**
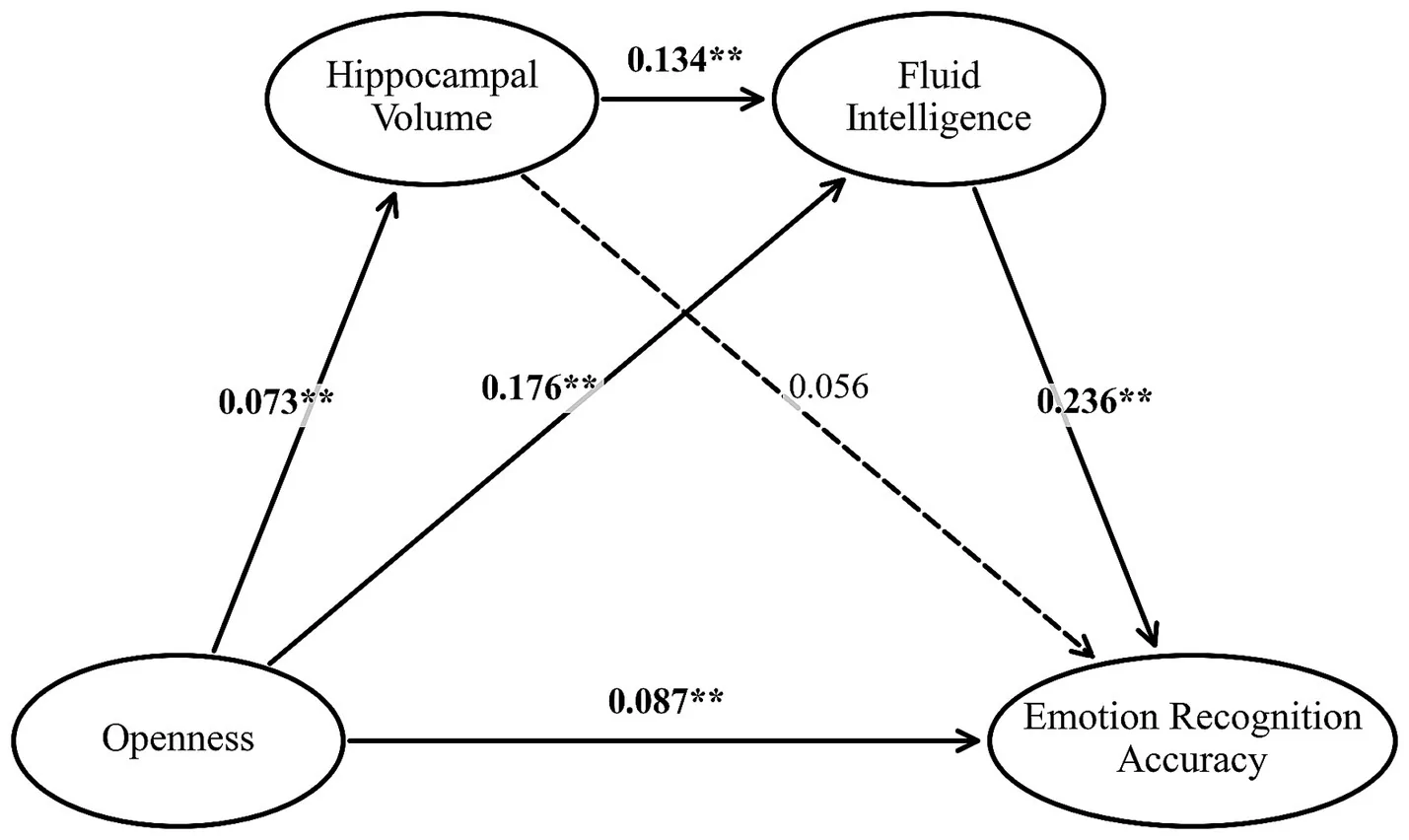
Results of the serial mediation model for emotion-recognition accuracy with standardized path coefficients. Solid lines = significant paths; dashed line = non-significant. Covariates are controlled but not shown. *p < 0.05, **p < 0.01, ***p < 0.001.

**Figure 4:**
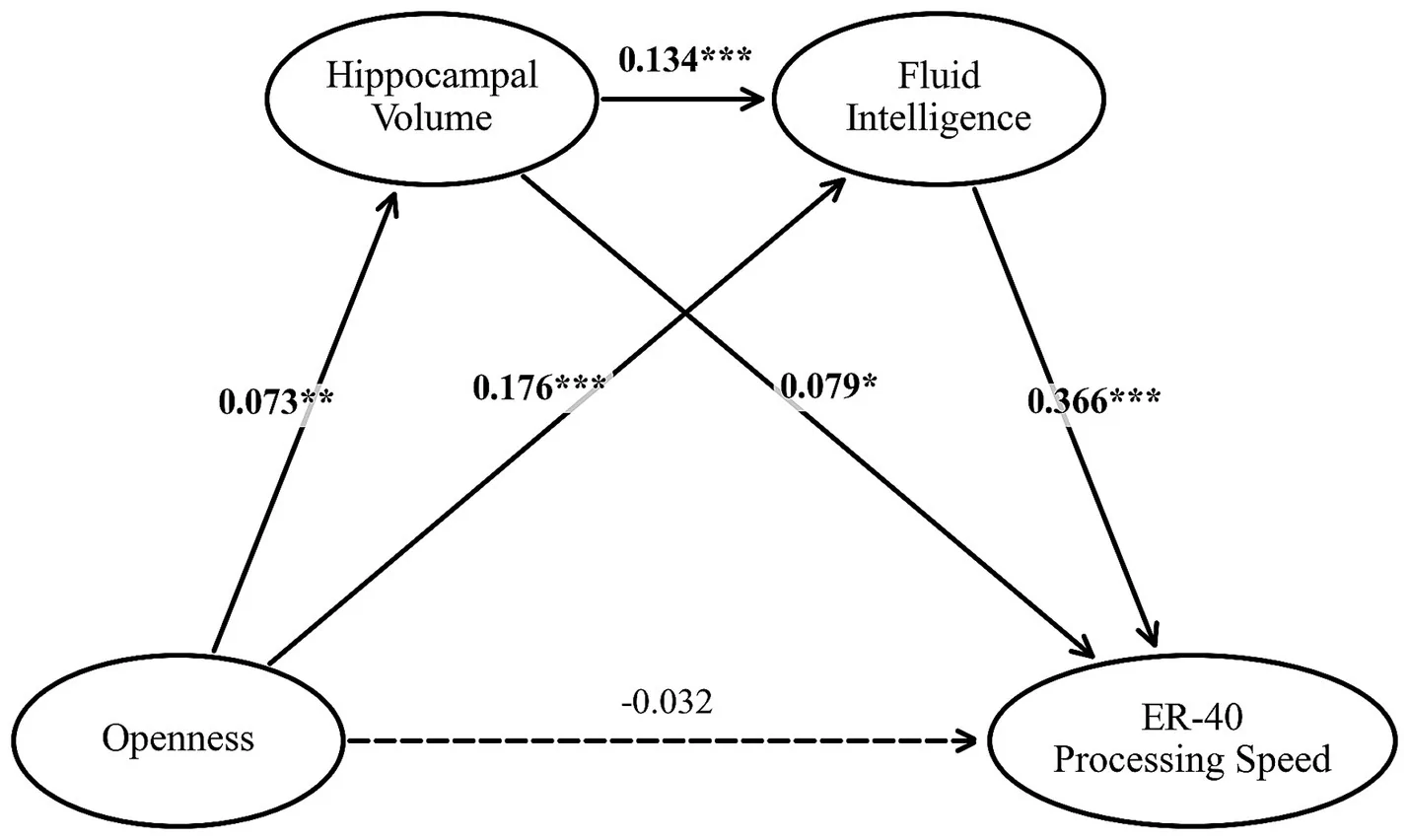
Serial mediation path coefficients for the processing speed model. Processing speed was operationalized as reaction time multiplied by −1 and standardized. Solid lines = significant paths; dashed line = non-significant. Covariates controlled but not shown. *p < 0.05, **p < 0.01, ***p < 0.001.

Under statistical control of age, sex, intracranial volume, and acquisition site, a serial mediation analysis was conducted using PROCESS Model 6, with openness as the independent variable, hippocampal volume as the first mediator, fluid intelligence as the second mediator, and the number of correct emotion-recognition responses and processing speed as the dependent variable in separate models. Standardized regression coefficients for each path are reported in Tables 3, 4, and the serial mediation models are shown in Figures 3, 4.

**Table 3:** Standardized path coefficients from the serial mediation model.

Path	*β*	SE	*t*	*p*
Openness → hippocampal volume	0.073	0.024	3.05	0.002
Openness → fluid intelligence	0.176	0.027	6.44	< 0.001
Hippocampal volume → fluid intelligence	0.134	0.038	3.55	< 0.001
Openness → ER-40 accuracy	0.087	0.032	2.76	0.006
Hippocampal volume → ER-40 accuracy	0.056	0.043	1.31	0.192
Fluid intelligence → ER-40 accuracy	0.236	0.038	6.23	< 0.001

All core variables *z*-standardized. Covariates (age, sex, intracranial volume, acquisition site) included in all equations but not displayed.

**Table 4:** Standardized path coefficients from the serial mediation model with ER-40 processing speed as outcome.

Path	*β*	SE	*t*	*p*
Openness → hippocampal volume	0.073	0.024	3.05	0.002
Openness → fluid intelligence	0.176	0.027	6.44	<0.001
Hippocampal volume → fluid intelligence	0.134	0.038	3.55	<0.001
Openness → ER-40 processing speed	−0.032	0.026	−1.24	0.215
Hippocampal volume → ER-40 processing speed	0.079	0.035	2.24	0.025
Fluid intelligence → ER-40 processing speed	0.366	0.031	11.79	<0.001

All core variables *z*-standardized. Processing speed = reverse-coded ER-40 reaction time (higher = faster). Covariates (age, sex, intracranial volume, acquisition site) included but not displayed. *N* = 912.

#### 3.3.1 Model path coefficients

The standardized regression coefficients for all paths of emotion-recognition accuracy are presented in Table 3. Openness significantly and positively predicted hippocampal volume (*β* = 0.073, SE = 0.024, *t* = 3.05, *p* = 0.002) and fluid intelligence (*β* = 0.176, SE = 0.027, *t* = 6.44, *p* < 0.001). Hippocampal volume significantly and positively predicted fluid intelligence (*β* = 0.134, SE = 0.038, *t* = 3.55, *p* < 0.001). Fluid intelligence significantly and positively predicted the number of correct emotion-recognition responses (*β* = 0.236, SE = 0.038, *t* = 6.23, *p* < 0.001). The direct effect of openness on the number of correct emotion-recognition responses also reached statistical significance (*β* = 0.087, SE = 0.032, *t* = 2.76, *p* = 0.006), indicating that openness still independently predicted emotion recognition performance after controlling for the two mediators. The path from hippocampal volume to the number of correct emotion-recognition responses did not reach significance (*β* = 0.056, SE = 0.043, *t* = 1.31, *p* = 0.192), suggesting that the effect of hippocampal volume on emotion recognition was transmitted mainly indirectly through fluid intelligence.

The standardized regression coefficients for the processing speed model are presented in Table 4. The upstream paths from openness to hippocampal volume and fluid intelligence, and from hippocampal volume to fluid intelligence, were consistent with the accuracy model. Fluid intelligence significantly predicted processing speed (*β* = 0.366, *p* < 0.001). Unlike the accuracy model, hippocampal volume showed a significant direct path to processing speed (*β* = 0.079, *p* = 0.025), whereas the direct effect of openness on processing speed was non-significant (*β* = −0.032, *p* = 0.215).

#### 3.3.2 Direct and indirect effects

The direct effect of openness on the number of correct emotion-recognition responses was significant (*β* = 0.087, SE = 0.032, *t* = 2.76, *p* = 0.006), indicating that openness still independently predicted emotion recognition performance after hippocampal volume and fluid intelligence were included as mediators.

Bias-corrected bootstrap analyses were used to further test the indirect effects; the results are shown in Table 5. The total indirect effect of openness on the number of correct emotion-recognition responses through the mediators was significant (Effect = 0.048, BootSE = 0.011, 95% CI [0.029, 0.070]). Examination of the three specific indirect paths showed the following. First, the indirect effect of openness on the number of correct emotion-recognition responses through fluid intelligence was significant (Effect = 0.041, BootSE = 0.010, 95% CI [0.024, 0.062]). This path accounted for the largest proportion of the total indirect effect, indicating that fluid intelligence was the primary mediating mechanism linking openness and emotion recognition. Second, the serial indirect effect of openness on the number of correct emotion-recognition responses sequentially through hippocampal volume and fluid intelligence was significant (Effect = 0.002, BootSE = 0.001, 95% CI [0.001, 0.005]). Because the 95% bootstrap confidence interval did not include 0, the serial mediation effect of openness on emotion-recognition accuracy through hippocampal volume and then fluid intelligence was supported. However, the magnitude of this serial indirect effect was modest, accounting for approximately 4% of the total indirect effect. This finding therefore indicates a statistically reliable but small neurocognitive pathway, and should not be interpreted as evidence of a dominant route through which openness influences emotion recognition. Third, the indirect effect of openness on the number of correct emotion-recognition responses through hippocampal volume alone was not significant (Effect = 0.004, BootSE = 0.004, 95% CI [−0.002, 0.012]). Because the 95% bootstrap confidence interval included 0, hippocampal volume could not independently mediate the effect of openness on emotion recognition.

**Table 5:** Bootstrap estimates of indirect effects of openness on ER-40 accuracy (Nboot = 5,000).

Indirect path	Effect	BootSE	BootLLCI	BootULCI
Total indirect effect	0.048	0.011	0.029	0.070
Openness → hippocampal volume → ER-40 accuracy	0.004	0.004	−0.002	0.012
Openness → fluid intelligence → ER-40 accuracy	0.041	0.010	0.024	0.062
Openness → hippocampal volume → fluid intelligence → ER-40 accuracy	0.002	0.001	0.001	0.005

CIs excluding zero indicate a significant indirect effect. All core variables *z*-standardized.

**Figure 5:**
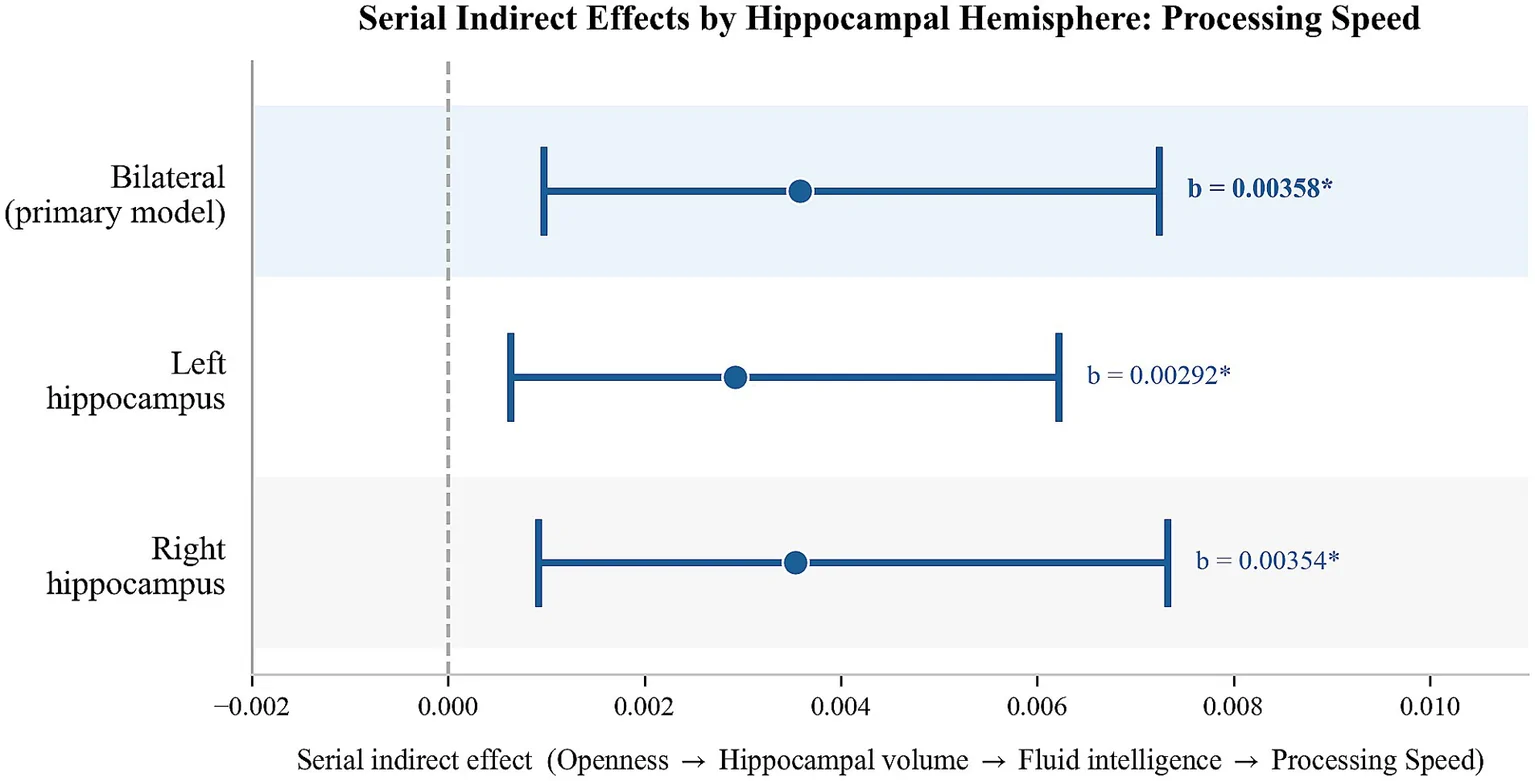
Serial indirect effects (Ind3) by hippocampal hemisphere for processing speed. Error bars = 95% bootstrap confidence intervals (5,000 resamples). * 95% CI excludes zero. *N* = 912.

Bootstrap analyses for the processing speed model are reported in Table 6. The serial indirect effect of openness on processing speed through hippocampal volume and fluid intelligence was significant (*b* = 0.004, 95% CI [0.001, 0.007]), replicating the pattern observed for accuracy. The indirect effect through fluid intelligence alone was also significant (*b* = 0.064, 95% CI [0.042, 0.091]), as was the indirect effect through hippocampal volume alone (*b* = 0.006, 95% CI [0.001, 0.013]; see Figures 4, 5).

**Table 6:** Bootstrap indirect effects from the serial mediation model with ER-40 processing speed as outcome.

Effect	*b*	Boot SE	Boot LLCI	Boot ULCI
Total indirect effect	0.074	0.013	0.051	0.100
Openness → hippocampal volume → ER-40 processing speed	0.006	0.003	0.001	0.013
Openness → fluid intelligence → ER-40 processing speed	0.064	0.012	0.042	0.091
Openness → hippocampal volume → fluid intelligence → ER-40 processing speed	0.004	0.002	0.001	0.007

All core variables *z*-standardized. Processing speed = reverse-coded ER-40 reaction time. Bootstrap SE and 95% CI based on 5,000 percentile resamples. CI not containing zero indicates significance. *N* = 912.

#### 3.3.3 Left and right hippocampal volume analyses

**Figure 6:**
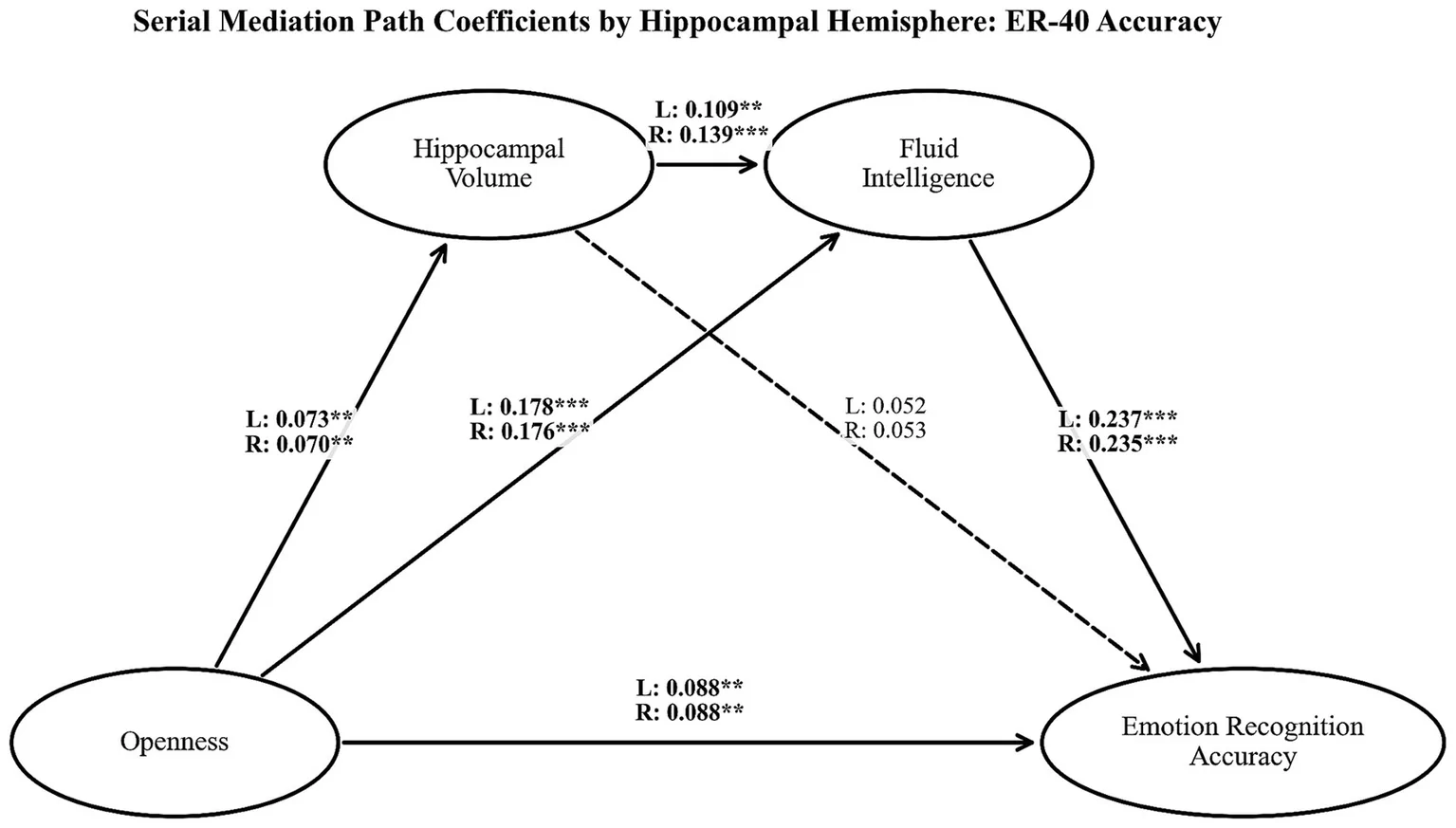
Serial mediation path coefficients for left (L) and right (R) hippocampal volume models (emotion-recognition accuracy outcome). Solid lines = significant paths; dashed line = non-significant. Covariates controlled but not shown. *p < 0.05, **p < 0.01, ***p < 0.001.

**Figure 7:**
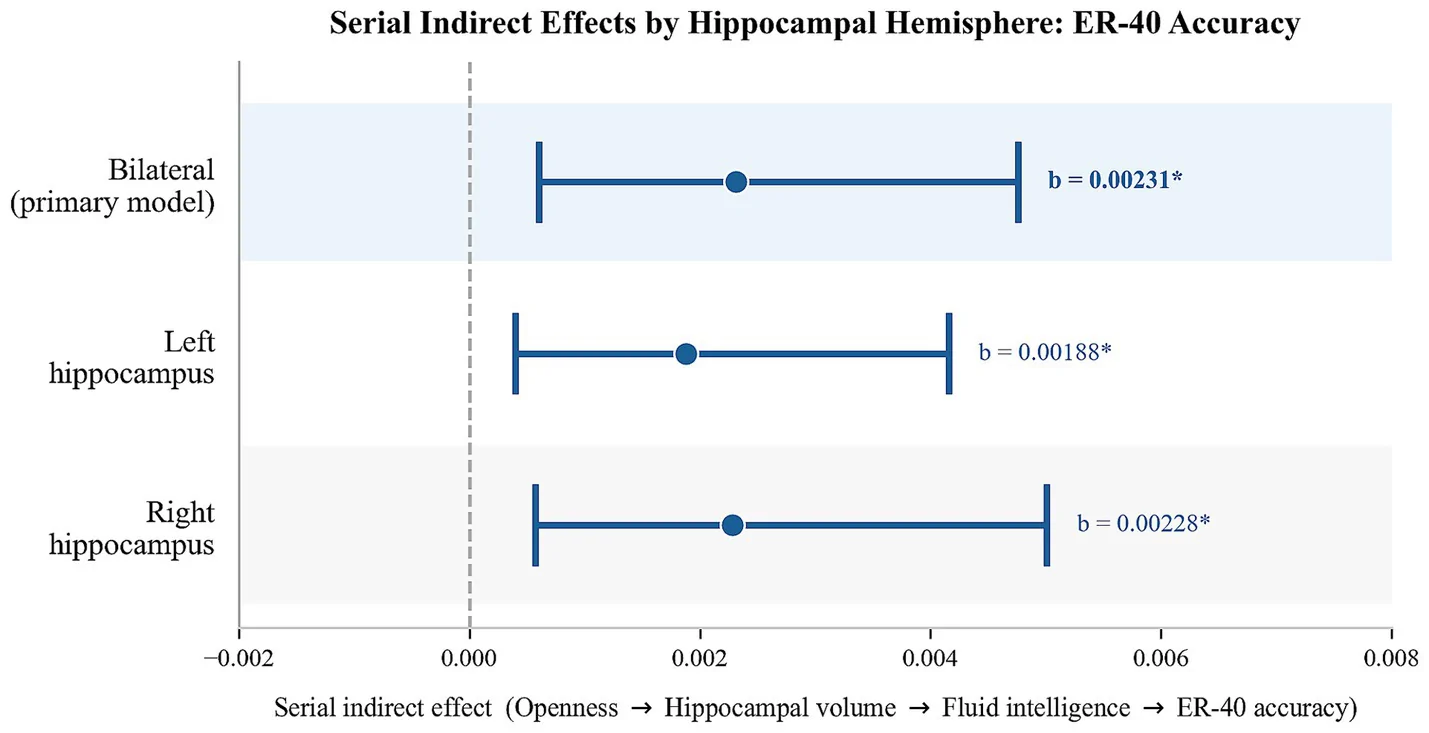
Serial indirect effects by hippocampal hemisphere for emotion-recognition accuracy. Error bars = 95% bootstrap confidence intervals (5,000 resamples). * 95% CI excludes zero. *N* = 912.

**Figure 8:**
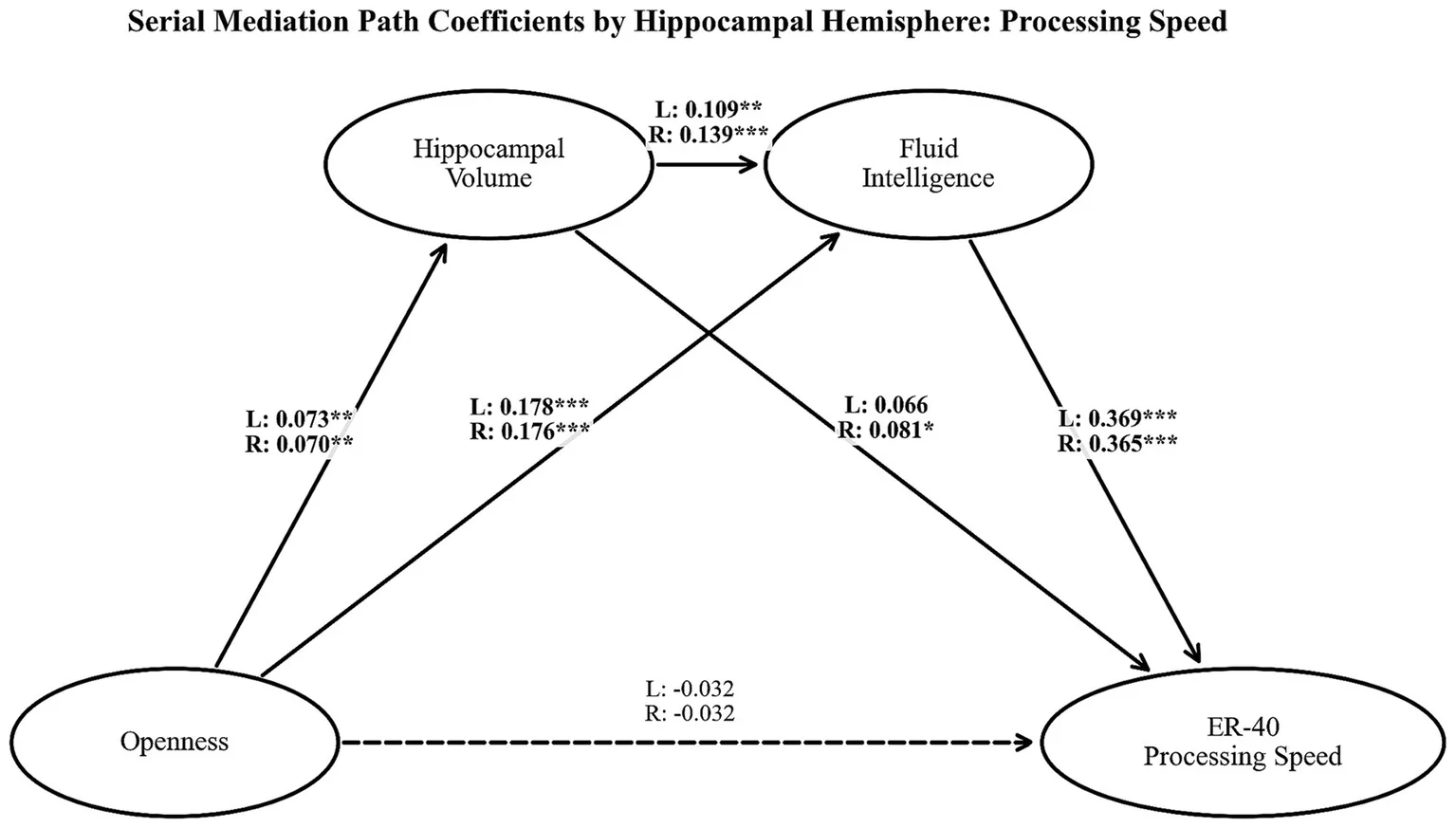
Serial mediation path coefficients for left (L) and right (R) hippocampal volume models (processing speed outcome). Solid lines = significant paths; dashed line = non-significant. Covariates controlled but not shown. *p < 0.05, **p < 0.01, ***p < 0.001.

To assess potential lateralization effects, the serial mediation model was rerun with left and right hippocampal volumes separately for both outcome variables. For emotion-recognition accuracy, the serial indirect effect remained significant for both left (Ind3 = 0.002, 95% CI [0.000, 0.004]) and right (Ind3 = 0.002, 95% CI [0.001, 0.005]) hippocampal volumes, and path coefficients were consistent with the primary model, suggesting the observed pathway was not hemisphere-specific (see Figures 6, 7). A parallel analysis using processing speed as the outcome yielded consistent results, with the serial indirect effect significant for both left (Ind3 = 0.003, 95% CI [0.001, 0.006]) and right (Ind3 = 0.004, 95% CI [0.001, 0.007]) hippocampal volumes (see Figures 5, 8).

### 3.4 Age moderation analysis

#### 3.4.1 Moderation model test

**Figure 9:**
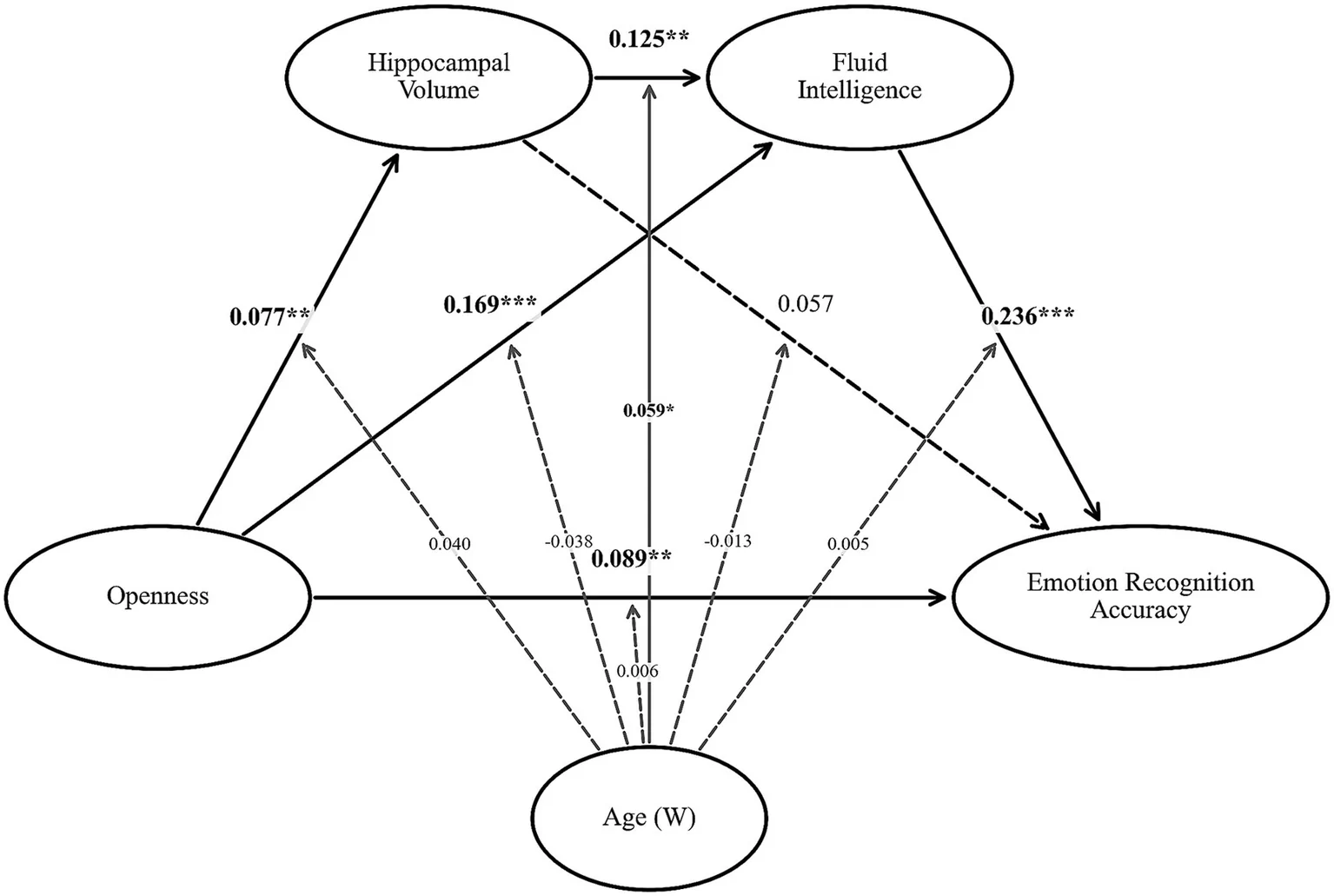
Age-moderated serial mediation model for emotion-recognition accuracy (PROCESS Model 92). Bold paths indicate significant moderation. Dashed lines indicate non-significant paths. Covariates omitted. *p < 0.05, **p < 0.01, ***p < 0.001.

Using PROCESS Model 92, age was entered as a continuous moderator of all paths in the serial mediation model simultaneously. Among all tested moderation paths, only the hippocampal volume-by-age interaction predicting fluid intelligence reached significance (*β* = 0.059, *p* = 0.030), indicating that the association between hippocampal volume and fluid intelligence was stronger at older ages. All other moderation paths were non-significant (all *p*s > 0.10; see Figure 9).

**Figure 10:**
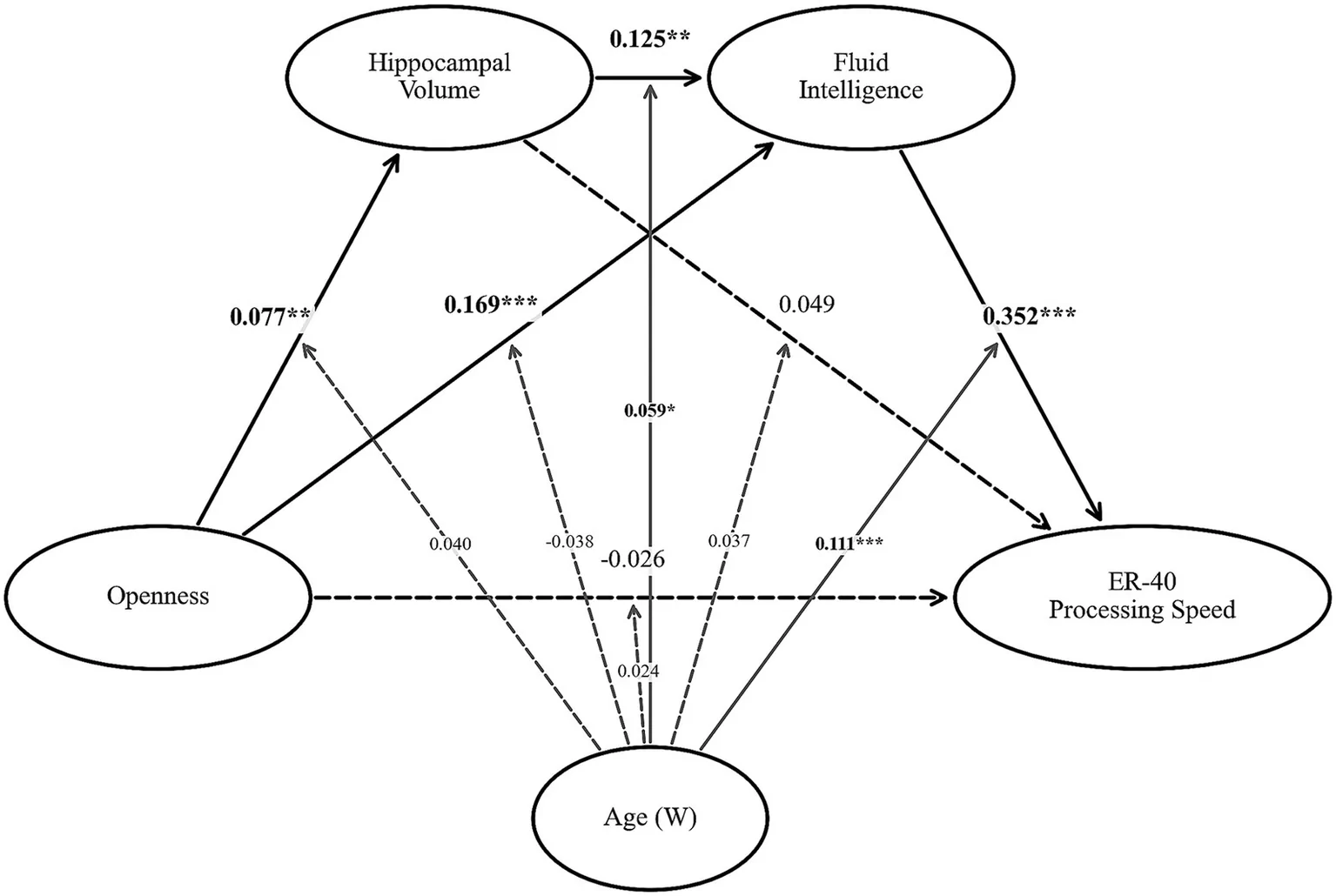
Age-moderated serial mediation model for processing speed (PROCESS Model 92). Bold paths indicate significant moderation. Dashed lines indicate non-significant paths. Covariates omitted. *p < 0.05, ***p < 0.001.

When processing speed was used as the outcome, the hippocampal volume-by-age interaction predicting fluid intelligence again reached significance (*β* = 0.059, *p* = 0.030). Additionally, the fluid intelligence-by-age interaction predicting processing speed was significant (*β* = 0.111, *p* < 0.001), indicating that the association between fluid intelligence and processing speed strengthened with advancing age (see Figure 10).

#### 3.4.2 Simple slopes analysis

**Figure 11:**
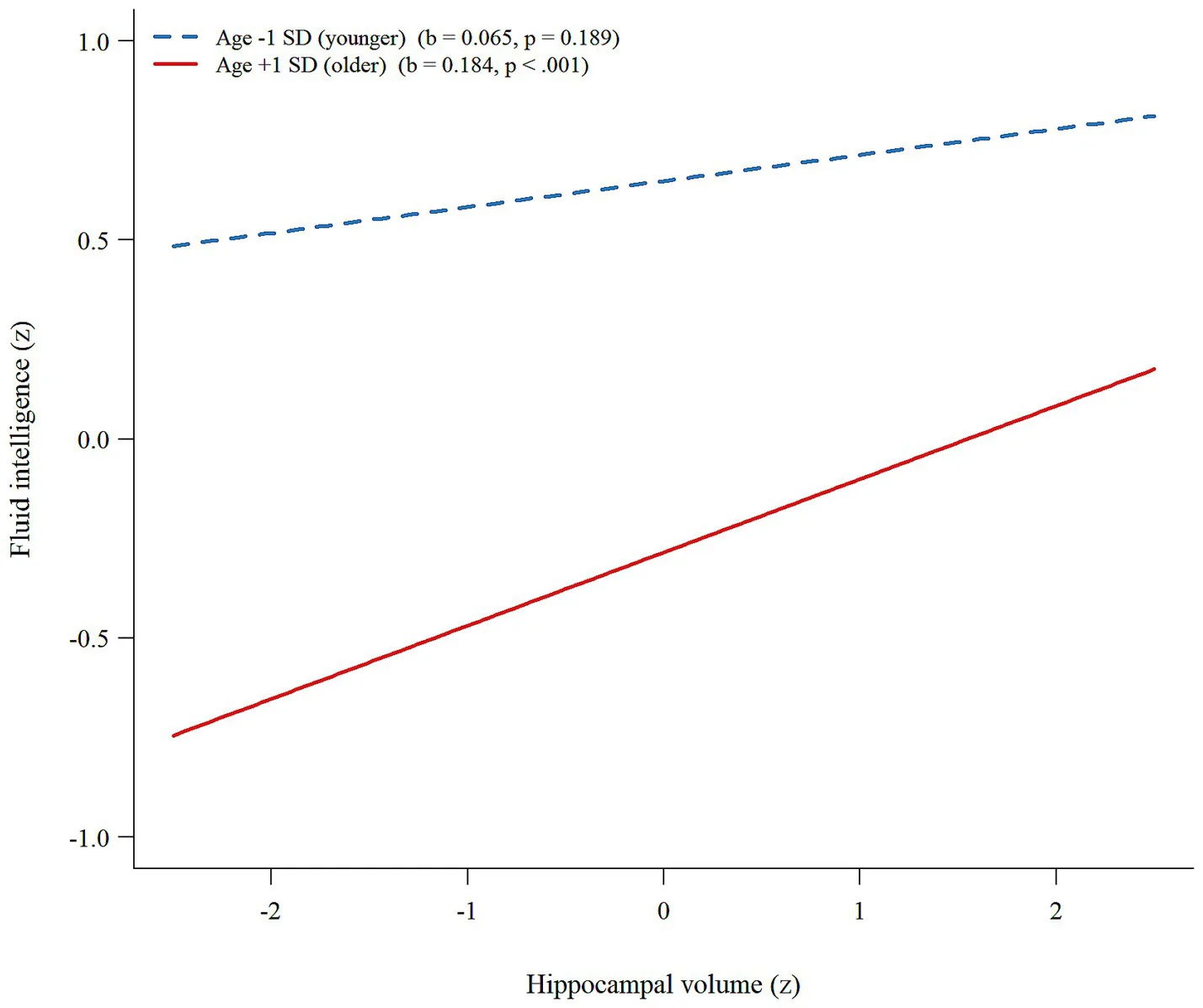
Simple slopes of the hippocampal volume-by-age interaction predicting fluid intelligence (emotion-recognition accuracy model). Regression lines represent the predicted association between hippocampal volume and fluid intelligence at lower and higher age levels. All variables were standardized prior to analysis.

Simple slope analysis of the significant hippocampal volume-by-age interaction revealed that the association between hippocampal volume and fluid intelligence was non-significant at lower age levels (*β* = 0.065, *p* = 0.189), but significant at mean age (*β* = 0.125, *p* = 0.001) and higher age levels (*β* = 0.184, *p* < 0.001). Correspondingly, the conditional serial indirect effect was significant at mean age (*b* = 0.002, 95% CI [0.001, 0.005]) and at higher age levels (*b* = 0.005, 95% CI [0.002, 0.011]), but not at lower age levels (*b* = 0.001, 95% CI [−0.001, 0.003]; see Figures 9, 11).

**Figure 12:**
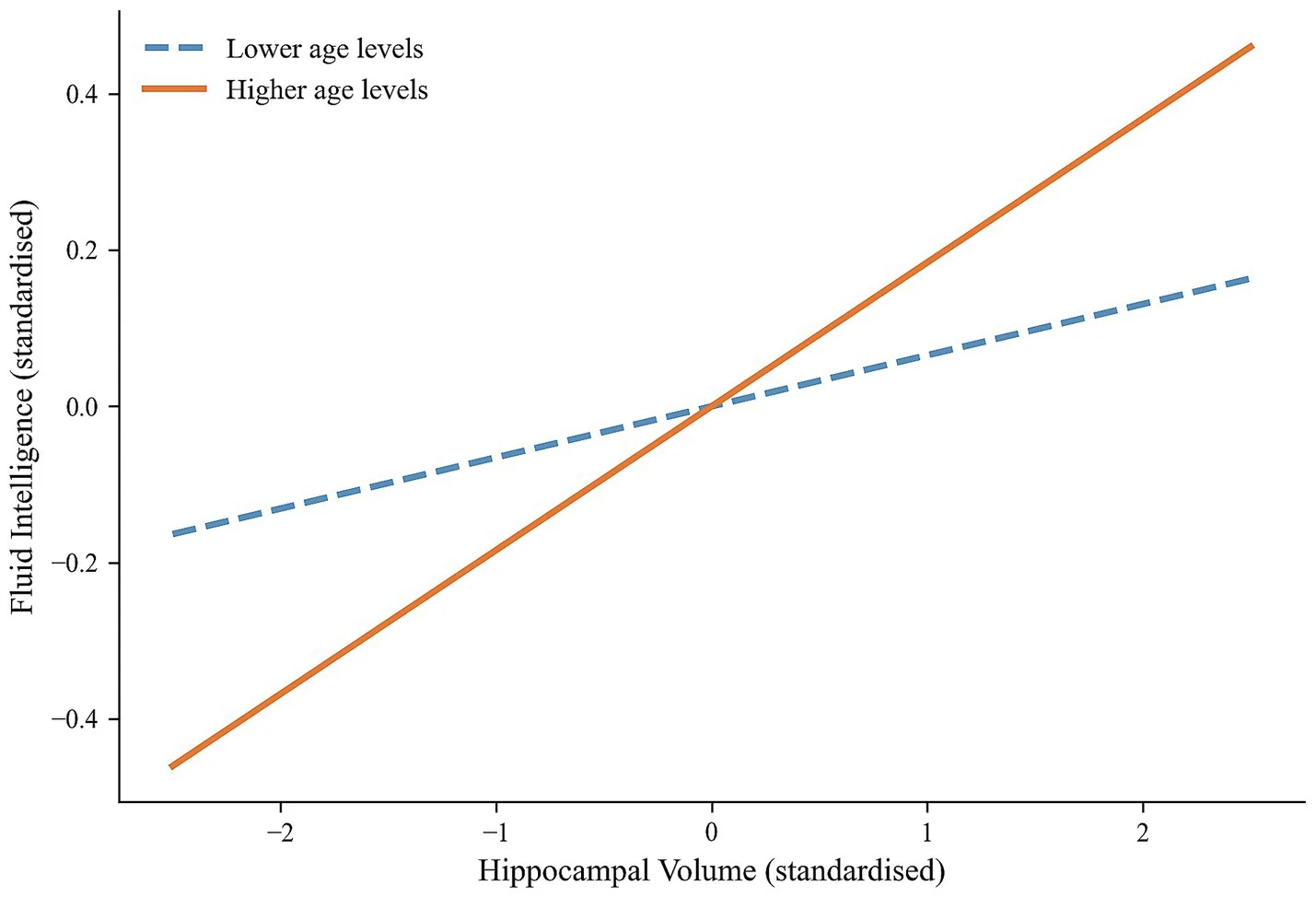
Simple slopes of the hippocampal volume-by-age interaction predicting fluid intelligence (processing speed model). Regression lines represent the predicted association between hippocampal volume and fluid intelligence at lower and higher age levels. All variables were standardized prior to analysis.

**Figure 13:**
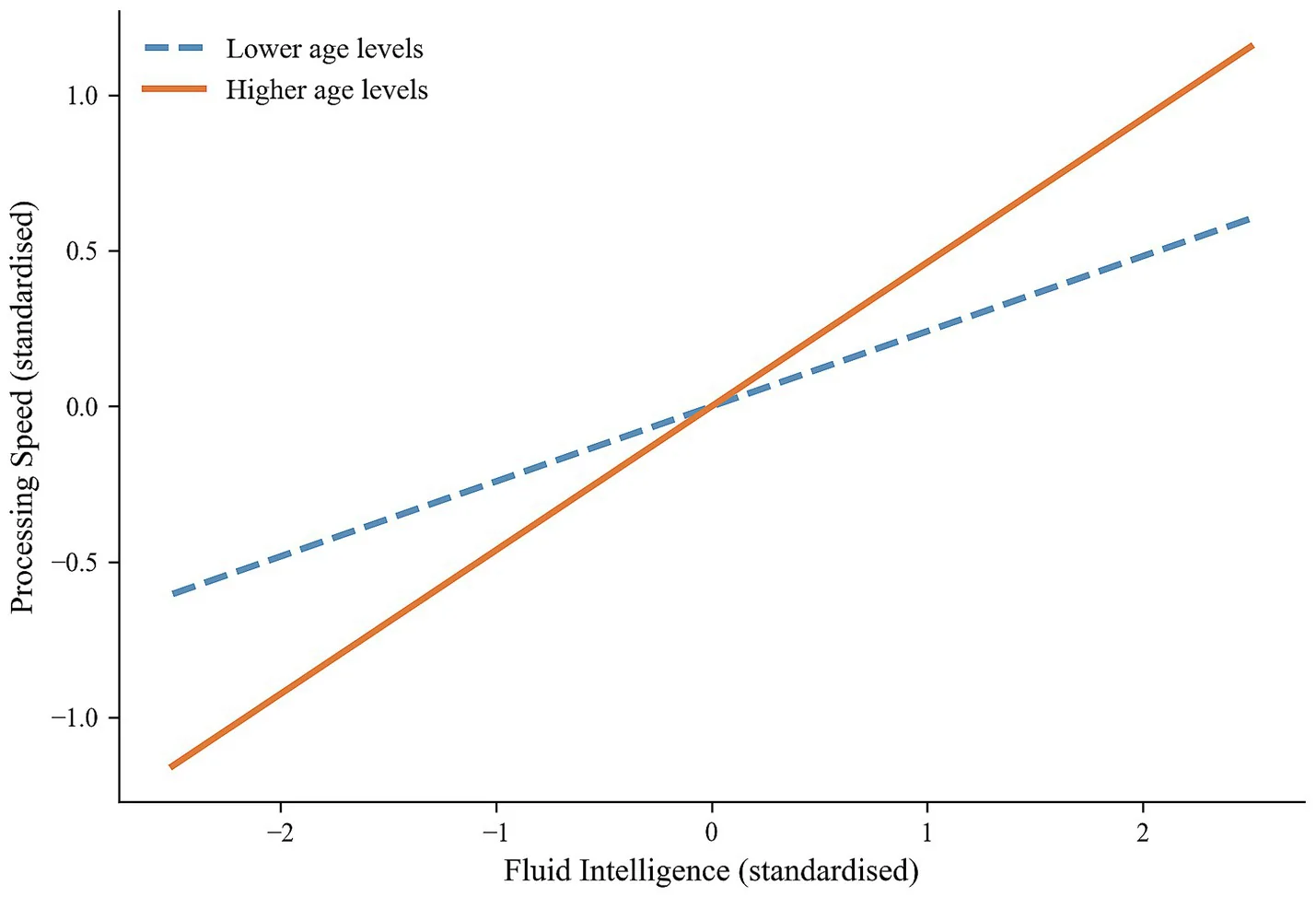
Simple slopes of the fluid intelligence-by-age interaction predicting processing speed. Regression lines represent the predicted association between fluid intelligence and processing speed at lower and higher age levels. All variables were standardized prior to analysis.

For the processing speed model, the same pattern of hippocampal volume-by-age slopes was observed (see Figure 12). Simple slope analysis of the fluid intelligence-by-age interaction further revealed that the association between fluid intelligence and processing speed strengthened monotonically with age (lower age levels: *β* = 0.241; mean age: *β* = 0.352; higher age levels: *β* = 0.462; all *p*s < 0.001; see Figure 13). The conditional serial indirect effect on processing speed was significant at mean age (*b* = 0.003, 95% CI [0.001, 0.007]) and higher age levels (*b* = 0.010, 95% CI [0.004, 0.019]), but not at lower age levels (*b* = 0.001, 95% CI [−0.001, 0.003]).

## 4 Discussion

The present study tested a serial mediation model in 912 middle-aged and older adults to examine how openness influences emotion recognition performance, operationalized as both accuracy and processing speed, through hippocampal volume and fluid intelligence. For emotion-recognition accuracy, the results supported Hypotheses 1, 3, and 4, but not Hypothesis 2. For processing speed, all four hypotheses were supported, as hippocampal volume showed a significant direct path to processing speed in addition to the serial indirect effect. The central finding is that the indirect effect of openness on emotion recognition relied almost entirely on fluid intelligence as the cognitive hub. Whether in the simple mediation path from openness through fluid intelligence or in the serial path from openness through hippocampal volume and fluid intelligence, fluid intelligence was an indispensable intermediate link. By contrast, the path from openness to emotion recognition through hippocampal volume alone was not significant. This pattern reveals a sequential neurocognitive pathway from personality traits to brain structure and then through domain-general cognitive resources to social cognitive performance, with fluid intelligence serving as the key translational mechanism through which structural brain preservation is converted into social cognitive advantage.

Traditional views regard the hippocampus as the core structure for declarative memory encoding and consolidation, with functions more closely related to crystallized rather than fluid intelligence. The significant path from hippocampal volume to fluid intelligence in the present study may therefore appear counterintuitive. However, cognitive dedifferentiation theory offers a compelling explanation. As the hippocampus undergoes pronounced age-related atrophy (24), its integrity increasingly becomes a shared neural bottleneck that constrains multiple cognitive functions simultaneously (40, 41). The present finding is consistent with prior evidence that the positive association between hippocampal volume and fluid intelligence is age-specific and spans multiple cognitive subdomains (30, 31). Moreover, the hippocampus supports relational binding, pattern separation, and flexible information retrieval, all of which overlap with core components of fluid intelligence. The NIH Toolbox Fluid Cognition Composite used in the present study also includes the Picture Sequence Memory Test, an episodic memory component heavily dependent on hippocampal function, further increasing the sensitivity of this index to hippocampal volume. Thus, the predictive effect of hippocampal volume on fluid intelligence is not inconsistent with its classical role, but rather reflects a principle of cognitive dedifferentiation in aging, whereby the constraining role of brain structural integrity on multiple cognitive functions becomes increasingly salient as neural resources diminish.

The present findings clarify a neurocognitive route through which hippocampal volume is associated with ER-40 performance in aging. In the accuracy model, the hippocampal contribution was fully mediated by fluid intelligence, with no significant direct path to ER-40 accuracy. Rather than indicating a direct structural basis for emotion perception, this pattern suggests that hippocampal volume is linked to emotion-recognition accuracy primarily through domain-general cognitive resources. These resources may support the controlled decoding of socially meaningful emotional information, particularly in middle-aged and older adults. Although the Introduction drew on the preliminary findings of Szymkowicz et al. (26) to propose a possible direct hippocampal contribution to emotion recognition, the present data indicate that this pathway is more parsimoniously explained through fluid cognitive capacity rather than a direct structural link. In contrast, when processing speed was used as the outcome, hippocampal volume showed a significant direct path, suggesting that hippocampal integrity may contribute more directly to the efficiency than to the accuracy of emotion-recognition responses. The CRUNCH model provides a theoretical framework for this interpretation (42). As processing efficiency declines, the aging brain recruits additional cognitive resources for compensation, but the effectiveness of this strategy is constrained by the available resource pool. Against this background, facial emotion recognition may become less automatic and more resource-demanding, making fluid intelligence an important bridge between neural structural integrity and social-cognitive performance. Across three independent studies, Connolly et al. (43) found that general intelligence significantly predicted cross-modal emotion recognition ability but did not predict facial identity recognition ability, further indicating that the contribution of intelligence to emotion recognition is specific to the cognitive decoding of emotional information. The systematic review and meta-analysis by Roheger et al. (44) point to a similar conclusion that social cognitive abilities, including emotion recognition and theory of mind, show progressive decline from healthy aging to Alzheimer’s disease, suggesting that social cognitive aging is intertwined with broader processes of cognitive deterioration. Fluid intelligence accounted for 85.4% of the total indirect effect, indicating that emotion recognition in middle-aged and older adults may depend on domain-general cognitive resources to a far greater extent than factor-analytic studies based on young samples would suggest. As processing efficiency declines in middle and later adulthood and compensatory recruitment of cognitive resources increases, the reliance of emotion recognition on fluid intelligence may deepen substantially. This age-related interpretation was supported by the moderation analyses and provides a more specific account of when the hippocampus-related pathway becomes relevant. From the perspective of cognitive dedifferentiation, the stronger hippocampal volume-fluid intelligence association at older ages suggests that structural integrity becomes increasingly consequential as compensatory neural resources decline. At relatively younger ages within the sample, residual cognitive reserve and neural redundancy may buffer against individual differences in hippocampal volume, weakening the structure-cognition association. With advancing age, this buffering capacity may diminish, making hippocampal integrity a more important constraint on fluid cognition. Consistent with this view, the serial indirect pathway was evident at mean and higher age levels but not at lower age levels, suggesting that the openness-hippocampus-fluid intelligence-emotion recognition pathway may be an aging-emergent route rather than a fixed association across adulthood. The processing-speed model extended this interpretation: the association between fluid intelligence and response speed also strengthened with age, indicating that fluid cognitive resources become increasingly important for efficient behavioral responding. Together, these findings suggest that the pathway from structural integrity to fluid cognition and ER-40 performance becomes more pronounced in later adulthood. This pattern also helps characterize potential vulnerability to social-cognitive decline: older individuals with lower openness, smaller hippocampal volume, or lower fluid intelligence may be more likely to show reduced emotion-recognition performance. Given the cross-sectional design, this interpretation should be understood as risk characterization rather than individual-level prediction.

Openness significantly and positively predicted hippocampal volume, and the supplementary correlation analyses provide further support for regional differences in this structural association. Zero-order correlations in Supplementary Table S1 showed that the correlation between openness and hippocampal volume (*r* = 0.160) was higher than that between openness and entorhinal cortex volume (*r* = 0.104). After controlling for age, sex, intracranial volume, and acquisition site, the partial correlations in Supplementary Table S2 became even clearer. Openness remained significantly correlated with hippocampal volume (*r* = 0.101, *p* = 0.002), whereas its partial correlation with entorhinal cortex volume was reduced to non-significance (*r* = 0.059, *p* = 0.076). This pattern is broadly consistent with the longitudinal findings of Giannakopoulos et al. (19), who reported that higher openness was associated with less left hippocampal volume loss. The reason why openness shows a stronger structural association with the hippocampus than with the entorhinal cortex still requires further study. Moreover, tau-related pathology in the entorhinal region is relatively common even in normal aging (38), which may weaken the sensitivity of entorhinal cortex volume to personality-related variation. At the same time, the dentate gyrus of the hippocampus retains some neurogenic capacity during aging, and this experience-dependent plasticity may make the hippocampus more likely to benefit from cognitively enriched lifestyles (45). Lateralization analyses further demonstrated that the serial indirect effect was significant for both left and right hippocampal volumes, suggesting that the observed pathway is not hemisphere-specific and that the bilateral mean volume provides a robust basis for characterizing this association.

After hippocampal volume and fluid intelligence were included in the model, the direct effect of openness on emotion recognition remained significant. This residual effect indicates that the proposed serial pathway is not the only route through which openness influences emotion recognition; an additional component may operate independently of brain structure and domain-general cognition. Parallel evidence suggests that after controlling for fluid intelligence and working memory, age-group differences in emotion recognition remained significant, indicating that emotion recognition retains a social cognitive component that is relatively independent of general cognition (29). The direct effect of openness may operate on precisely this component. Individuals higher in openness may be more likely to attend to and actively interpret others’ emotional expressions, and the accumulated perceptual experience gained from everyday social interaction may help preserve social perceptual ability in a manner that is less dependent on domain-general cognitive efficiency. Accordingly, the serial pathway identified in the present study should be understood as a major neurocognitive mechanism through which openness affects emotion recognition, rather than the only mechanism.

Taken together, the theoretical contribution of the present study lies in identifying an interpretable serial neurocognitive pathway through which personality traits influence social cognition and in clarifying the functional role of each component in this pathway. Fluid intelligence as the primary mediator indicates that emotion recognition in middle-aged and older adults is constrained to a considerable extent by domain-general cognitive resources, rather than being determined solely by decline in social-affective brain regions, such as the amygdala, fusiform gyrus, and anterior insula. The residual direct effect of openness, in turn, preserves a social cognitive component that is relatively independent of general cognition. The coexistence of these two components suggests that individual differences in emotion recognition depend both on domain-general cognitive infrastructure and on domain-specific socioemotional processes. From the perspective of cognitive reserve theory, the present study extends the cognitive protective effect of openness from the domains previously emphasized, namely fluid intelligence and memory, to the domain of social cognition, and shows that this effect is mediated through identifiable neurocognitive pathways (46).

At a practical level, the present findings suggest that prevention-oriented strategies for older adults may benefit from monitoring not only general cognitive decline but also social-cognitive functions such as emotion recognition. The moderated pathway indicates that vulnerability is more pronounced in later adulthood, particularly among individuals with lower openness, smaller hippocampal volume, or lower fluid intelligence. These characteristics may help identify groups who could benefit from closer monitoring or earlier cognitive and social-cognitive support. From a screening perspective, openness assessment may offer a low-cost and time-efficient first step for identifying older adults who may warrant closer follow-up. Individuals with lower openness could be regarded as a potentially vulnerable subgroup, for whom repeated assessment of fluid intelligence may provide a practical behavioral marker of neurocognitive vulnerability and possible risk for later emotion-recognition decline. The mediating role of fluid intelligence is particularly important in this context. Because repeated MRI assessment is costly and less feasible for broad screening, fluid-cognition measures may offer a more scalable behavioral indicator of vulnerability in hippocampus-related pathways. At the same time, because fluid intelligence lies on the pathway linking neural structural integrity to social-cognitive performance, individuals showing rapid decline in fluid cognition may be prioritized for future intervention studies using cognitive training, cognitive enrichment, or socially engaging activities to buffer downstream risks for emotion-recognition performance and psychological well-being.

The present study has several limitations. First, the cross-sectional design means that the ordering of variables in the model reflects only a theory-driven statistical pathway and cannot be interpreted as a strict temporal causal sequence; the temporal assumptions of the model require testing in longitudinal designs. Second, the emotion-recognition indicator was limited to the ER-40 total correct score. In middle-aged and older adults, impairments in recognizing negative emotions such as fear, anger, and sadness may be substantially greater than impairments in recognizing happy faces, and different emotion categories may vary in their dependence on cognitive resources. Use of a total score therefore cannot determine whether the mediating effect of fluid intelligence is more pronounced for emotion categories with higher cognitive demands, making emotion-specific analyses a priority for future work. Third, the NEO-FFI used in the present study measures only the overall openness score and cannot distinguish potentially differential contributions of subcomponents such as imagination, aesthetic experience, openness to feelings, and openness to ideas. Future research may use facet-level assessment with the NEO-PI-R to obtain a more fine-grained map of personality-social cognition relationships. Fourth, hippocampal volume was indexed as the bilateral mean, which obscures subregional differences and lateralization effects. Future studies may use hippocampal subfield segmentation techniques for more detailed investigation.

In summary, the present study revealed a serial indirect pathway through which openness influences emotion recognition through hippocampal volume and fluid intelligence. Within this pathway, fluid intelligence is the key hub through which protective effects at the level of brain structure are translated into social cognitive advantage, whereas the contribution of hippocampal volume is realized entirely indirectly through fluid intelligence. These findings indicate that individual differences in emotion recognition among middle-aged and older adults are rooted to a considerable extent in the availability of domain-general cognitive resources, and that the protective effect of openness on social cognition follows a continuous transmission chain from personality trait to brain structural integrity, then to cognitive resources, and finally to social cognitive performance. The generalisability of this pathway to processing speed further revealed that hippocampal volume contributes to emotion-recognition speed via an additional direct route, and that age amplifies both the hippocampal-fluid intelligence link and the fluid intelligence-speed link, suggesting a progressively tighter neurocognitive chain with advancing age.

## Data Availability

The original contributions presented in the study are included in the article/Supplementary material, further inquiries can be directed to the corresponding author.
