# Supplementary material for: Openness influences emotion recognition in middle-aged and older adults through hippocampal volume and fluid intelligence: a serial mediation model
Source: Front Public Health. 2026 Aug 13;14:1847936. doi: 10.3389/fpubh.2026.1847936 (PMC13520836; doi:10.3389/fpubh.2026.1847936)
Supplement: Supplementary file 1 [file Supplementary_file_1.docx]

**Supplementary Material**

**Table S1.** Correlations among hippocampal volume, entorhinal cortex volume, and openness

| Variable | M (SD) | 1 | 2 | 3 |
| --- | --- | --- | --- | --- |
| 1. Hippocampal volume (mm³) | 3841.61 (468.09) | — |  |  |
| 2. Entorhinal cortex volume (mm³) | 785.14 (135.74) | .410*** | — |  |
| 3. Openness | 29.85 (5.92) | .160*** | .104** | — |
|  |  |  |  |  |
| *Note. ** p < .01. *** p < .001. Age, sex, ICV, and site not controlled.* | | | | |

**Table S2.** Partial correlations among openness, hippocampal volume, and entorhinal cortex volume

| Variable | 1 | 2 | 3 |
| --- | --- | --- | --- |
| 1. Openness | — |  |  |
| 2. Hippocampal volume | .101** | — |  |
| 3. Entorhinal cortex volume | .059 | .248*** | — |
|  |  |  |  |
| *Note. ** p < .01. *** p < .001.* | | | |

**Table S3.** sensitivity analysis additionally controlling for race, ethnicity, and education

| Path | Effect | Boot SE | Boot LLCI | Boot ULCI |
| --- | --- | --- | --- | --- |
| Total indirect effect | 0.02090 | 0.00770 | 0.00690 | 0.03740 |
| Openness → hippocampal volume → ER-40 accuracy | 0.00250 | 0.00300 | -0.00250 | 0.00950 |
| Openness → fluid intelligence → ER-40 accuracy | 0.01700 | 0.00710 | 0.00460 | 0.03230 |
| Openness → hippocampal volume → fluid intelligence → ER-40 accuracy | 0.00140 | 0.00080 | 0.00020 | 0.00330 |
| *Note. All core variables z-standardised. Additional covariates: race, ethnicity (Hispanic/Latino vs. not), and years of education (continuous), alongside age, sex, intracranial volume, and acquisition site. Participants with unknown or not reported race were excluded (n = 20); analytic sample N = 892. Bootstrap 95% CI based on 5,000 percentile resamples.* | | | | |
| **Table S4.** sensitivity analysis additionally controlling for race, ethnicity, and education Path \| β \| SE \| t \| p; Openness → hippocampal volume \| 0.058 \| 0.025 \| 2.31 \| .021; Openness → fluid intelligence \| 0.081 \| 0.027 \| 2.94 \| .003; Hippocampal volume → fluid intelligence \| 0.115 \| 0.036 \| 3.16 \| .002; Openness → ER-40 accuracy \| 0.078 \| 0.033 \| 2.36 \| .018; Hippocampal volume → ER-40 accuracy \| 0.043 \| 0.044 \| 0.98 \| .328; Fluid intelligence → ER-40 accuracy \| 0.211 \| 0.041 \| 5.21 \| < .001; *Note. All core variables z-standardised.* \| \| \| \| | | | | |
|  | | | | |

**Figure S1.** Distribution of ER-40 accuracy scores (N = 912). Bars are colour-coded by distance from the sample mean: dark blue = below −1 SD (≤ 30); light blue = within ±1 SD (31–37); orange = above +1 SD (≥ 38). The dashed vertical line indicates the sample mean (M = 33.55, SD = 3.47).

**Figure S2.** Distribution of ER-40 reaction time (ms; N = 912). Bars are colour-coded by distance from the sample mean: dark blue = below −1 SD (< 1,642 ms); light blue = within ±1 SD (1,642–3,318 ms); orange = above +1 SD (> 3,318 ms). The dashed vertical line indicates the sample mean (M = 2,479.81 ms, SD = 837.95 ms).

**Figure S3**. Serial indirect effects of openness on ER-40 accuracy through alternative brain regions. Each region was tested as an independent structural mediator in place of hippocampal volume. Error bars indicate 95% bootstrap confidence intervals. The diamond marker indicates the only significant effect. * 95% CI excludes zero. N = 912.

**Figure S4**. Serial indirect effects of openness on processing speed through alternative brain regions. Each region was tested as an independent structural mediator in place of hippocampal volume. Processing speed = reaction time multiplied by −1 and standardised. Error bars indicate 95% bootstrap confidence intervals (5,000 resamples). * 95% CI excludes zero. N = 912.

**Figure S5.** Distributions of key variables (N = 912). Each panel shows a half-violin density plot, a box-and-whisker plot, and jittered raw data points for openness, hippocampal volume, fluid intelligence, and ER-40 accuracy. The red diamond indicates the mean.
